# Tetany

**Published:** 1891-05

**Authors:** E. H. Small

**Affiliations:** Pittsburg, Pa.


### TETANY.*

By E. H. SMALL, M. D., PITTSBURG, PA.

On November 29th, 1890, I was called to see M. B., a fat, healthy-looking, breast-fed baby boy, aged 11 months. His hands and feet were much swollen, œdematous, and of a cyanotic tinge. His mother said that they had been "spotted," *i. e.*, ecchymotic, before I had come. The fingers were strongly flexed at the metacarpal phalangeal joints, while the phalangeal

*Read before the Allegheny County Medical Society, March 17, 1891.

joints were as strongly extended. The thumbs were adducted and flexed. The feet were extended at the ankles as in talipes equinus, while the toes were strongly flexed. Attempts to straighten out these contractions caused great pain. The mother said that at first the child cried a good deal, and that his hands and feet were tender and painful. So much were the hands and feet, particularly the dorsal surface, swollen, that I suspected nephritis. The urine, however, contained no albumen. The child had always been strong and healthy, having had no other sickness.

Two days before, Thanksgiving, the child had been given some turkey and cranberries to eat, which had caused indigestion. When I called he had had no satisfactory movement of the bowels for some time. I gave him two one-half grain doses of calomel one hour apart, and twenty drops of the elixir of bromide of potassium four times daily, and told the mother to rub his hands and feet with alcohol and water. The next day he was much better. I directed hot fomentations to be applied to the hands and feet instead of the rubbing with alcohol and water. In a week the child was about as well as usual.

Four weeks before his gums had been scored by another doctor. A few days before my visit, the two first teeth had appeared, *i. e.*, at eleven months. The anterior fontanelle was larger than normal for his age, and the costo-chondral articulations were rather more prominent than normal. He has an older brother and sister who are perfectly healthy.

When I first saw this case, I thought it to be one of tetany, and its course and termination have proved it to have been such.

I had never before seen this disease in a child, but had seen one case in Vienna in a pregnant woman.

Although this disease has doubtless always existed, and although it was described as far back as 1831 by a Frenchman, M. Dance, as occurring in an adult, and in 1832 by another Frenchman, M. Tonnele, as a new convulsive disease of childhood, yet it is but seldom mentioned in the more common medical text-books. The name tetany was first given it by Dr. Corvisart, in 1851. Dunglison's Dictionary, 1874, speaks of "Tetanilla" diminutive of tetanus, saying that this disease is also called tetany.

Dr. Smith, of New York, defines it as "a disease in which there is a tonic contraction of the muscles, commonly those of the extremities, but sometimes also those of the face or trunk, produced by causes external to the nervous system, and usually of temporary duration." This definition shuts out true muscular contractions arising from disease of the brain or spinal cord, in which the contractions are both but a symptom, and not the disease itself. Henoch describes it under the name of "Idiopathischen contracturen" and regards it as a kind of abortive form of convulsions. Dr. Cherdle, of London, says, "Laryngismus, tetany and general convulsions are the positive, comparative and superlative of the convulsive state in childhood."

CAUSES.—Cases are recorded between the ages of six months and sixty-one years. Most cases occur in infancy and childhood; more in males than in females. The most common cause seems to be disorders of the digestive system, as diarrhœa, habitual constipation, worms, and dentition. Charles Warrington Earle, of Chicago, gives a case of a healthy girl two and one half years old, in whom tetany occurred on the day after she had eaten heartily of fried potatoes. Perhaps my case was caused by the turkey and cranberry sauce of Thanksgiving, two days before.

It may arise in persons who are in poor health from other diseases, as pneumonia, bronchitis, cholera, typhoid fever and dysentery. Exposure to wet and cold has seemed to cause it. Hence some think it a rheumatic affection. Erb says: "Many physicians have regarded it as an exquisite example of rheumatic disease." In adults, commencing puberty, pregnancy, as in the case I saw in Vienna, and nursing, may cause it. Rachitis is also regarded as a cause, which may hold in my case, on account of the delayed dentition, large size of fontanelle and enlarged articulations.

SYMPTOMS.—In patients old enough to describe their symptoms, tetany begins with pain in the head and an uneasy, tingling, burning sensation in the limbs. In children, the objective symptoms are those first noticed. The peculiar shape of the hands and feet, their rigidity, and pain on pressure are the commonest symptoms. Generally the fingers and toes are flexed on the palms and soles, occasionally extended. At times the joints of the hands and feet are also affected, or the elbow-joint—so that the fore-arm appears flexed upon the humerus, the hands upon the fore-arm, and the foot upwards, or else towards the sole. The thighs may be adducted, or flexed, the legs extended or flexed, and the feet extended as in talipes equinus. The contractions are always bilateral and symmetrical. Attempts to straighten out the contractions cause pain. Œdema, with a cyanotic tinge of the back of the hands and feet and occasionally ecchymoses, produced according to Henoch, by the pressure of the contracted muscles on the intermuscular veins, is ofttimes present. In severe cases the muscles of the trunk and head may be affected, but this is rare in children. Trousseau's sign—compression of the artery and nerve supplying the contracted muscles increasing the contractions—can be sometimes observed. The electrical excitability of the nerve supplying the affected muscles is increased, as is also the patellar reflex.

DIAGNOSIS.—This may be made out by the peculiar grouping of the symptoms, the characteristic position of the extremities and the absence of cerebral and general disturbances. Tetanus neonatorum and organic disease of the brain and spinal cord are the principal diseases with which it may be confounded. Tetanus generally occurs within a few days after birth, almost never after the first month ; tetany is very rare under the age of one month. In tetanus the muscles of mastication are early affected ; in tetany the contractions begin in the extremities, and the muscles of mastication are never, or only in the last stages, affected. In tetanus the symptoms tend rapidly to become worse and worse, generally ending in death; in tetany, as a rule, the child is soon well. Tetanus is in some way connected with injury to the umbilicus, or umbilical cord; in tetany trauma has nothing to do with the case. In organic diseases of the brain the contractions are usually limited to one side, with other symptoms of brain involvement; in tetany the contractions are bi-lateral.

PROGNOSIS.—In children tetany, when uncomplicated by grave disease causing it, almost always ends in recovery, though it may recur. The duration is from a few days to several weeks or months—indefinite.

PATHOLOGY.—Since tetany in children is so rarely fatal, and then usually from the complicating or causative disease, but few autopsies have been made, and in these no lesions have been found which seem to bear a causal relation to the disease. Herz says that clinical phenomena indicate that the disease is due to anæmia of the cord.

TREATMENT.—When the cause is known, especially when from disease of the digestive system, its removal will soon be followed by the disappearance of the disease. Bromide of potassium in doses according to age should be used. Chloral and calabar bean are recommended. Envelop the hands and feet in hot fomentations ; or use massage with alcohol and water. A child of fifteen months recovered in one week on gr. $\frac{1}{4}$ zinc sulphate and gr. $\frac{1}{192}$ atrophia sulphate, thrice daily. This is all that is necessary in children. In adults canabis indica and morphia hypodermically have been used with good results.

